# Assessment and diagnosis of chronic dyspnoea: a literature review

**DOI:** 10.1038/s41533-022-00271-1

**Published:** 2022-03-08

**Authors:** Anthony Paulo Sunjaya, Nusrat Homaira, Kate Corcoran, Allison Martin, Norbert Berend, Christine Jenkins

**Affiliations:** 1 Respiratory Group, The George Institute for Global Health, Sydney, NSW Australia; 2 Discipline of Paediatrics, School of Women’s and Children’s Health, Faculty of Medicine, UNSW Sydney, Sydney, NSW Australia; 3 Respiratory Department, Sydney Children’s Hospital, Randwick, Sydney, NSW Australia; 4 Faculty of Medicine, UNSW Sydney, Sydney, NSW Australia; 5 Department of Thoracic Medicine, Concord Hospital, Concord, Sydney, NSW 2139 Australia

**Keywords:** Respiratory signs and symptoms, Physical examination

## Abstract

Dyspnoea or breathlessness is a common presenting symptom among patients attending primary care services. This review aimed to determine whether there are clinical tools that can be incorporated into a clinical decision support system for primary care for efficient and accurate diagnosis of causes of chronic dyspnoea. We searched MEDLINE, EMBASE and Google Scholar for all literature published between 1946 and 2020. Studies that evaluated a clinical algorithm for assessment of chronic dyspnoea in patients of any age group presenting to physicians with chronic dyspnoea were included. We identified 326 abstracts, 55 papers were reviewed, and eight included. A total 2026 patients aged between 20–80 years were included, 60% were women. The duration of dyspnoea was three weeks to 25 years. All studies undertook a stepwise or algorithmic approach to the assessment of dyspnoea. The results indicate that following history taking and physical examination, the first stage should include simply performed tests such as pulse oximetry, spirometry, and electrocardiography. If the patient remains undiagnosed, the second stage includes investigations such as chest x-ray, thyroid function tests, full blood count and NT-proBNP. In the third stage patients are referred for more advanced tests such as echocardiogram and thoracic CT. If dyspnoea remains unexplained, the fourth stage of assessment will require secondary care referral for more advanced diagnostic testing such as exercise tests. Utilising this proposed stepwise approach is expected to ascertain a cause for dyspnoea for 35% of the patients in stage 1, 83% by stage 3 and >90% of patients by stage 4.

## Introduction

Dyspnoea or breathlessness is a complex symptom deriving from interactions of physiological, psychological, social and environmental factors and can only be perceived “by the person experiencing it”^1,2^. It has many causes and may present as sudden onset or more sub-acutely, with many years of progressively worsening symptoms^3–5^. Among this latter group, the most common diagnoses have a respiratory or cardiac origin and include diseases such as asthma, chronic obstructive pulmonary disease (COPD)^6^ and heart failure (HF). As populations become more sedentary and overweight, and retirement age increases, dyspnoea may increase in frequency and impact productivity, healthcare usage, independence and demand for community services^7,8^.

Environmental effects are an emerging area of interest contributing to dyspnoea. Changes to the biosphere and environment due to climate change will likely lead to an increased frequency of extreme weather events such as bushfires^9,10^, heatwaves and colder winters, all of which negatively affect cardiopulmonary health^11^. Furthermore, the yet unknown long-term sequelae of coronavirus disease 2019 (COVID-19) for the millions that have been affected, are expected to further increase the burden of dyspnoea and presentations to healthcare professionals^12^.

Many of the medical and lifestyle problems which contribute to dyspnoea are treatable. However, misdiagnosis or incorrect attribution of cause can result in suboptimal symptom control, overuse of pharmaceuticals, potentially serious side effects, and excessive cost to patients and the health system^13^. As an example, in a study using questionnaires and spirometry to estimate the burden of obstructive lung disease in urban and regional Australia, 29% of people who said a doctor had diagnosed COPD, emphysema or chronic bronchitis, actually had no evidence of airflow limitation^14^. This apparent over-diagnosis was matched by similar levels of under-diagnosis. In this same study, the prevalence of shortness of breath when hurrying or climbing a slight hill was 25.2% (95% CI, 22.7–27.6%) demonstrating the high prevalence of dyspnoea in Australians aged over 40 years. Similarly, in Italy, it was reported that only one-third (30.7%) of participants with daily respiratory symptoms had undergone any lung function tests. Moreover, the prevalence of self-reported physician diagnosis was 1.4%, far lower than the 9.1% to 11.7% prevalence based on spirometry^15^.

In addition to wasted opportunity to prevent morbidity and address lifestyle issues, inappropriate prescription of medication and expensive testing is often undertaken to exclude serious disease^16^. Conversely, if accurate diagnosis and appropriate management of dyspnoea could be hastened, the risk of untreated disease and comorbid illness would be reduced, and hence healthcare costs^17^. More accurate, systematic evaluation and management of patients with chronic breathlessness has the potential to improve quality of life and reduce work absenteeism, premature retirement, healthcare costs and productivity loss.

This narrative review aims to provide a comprehensive overview of validated clinical algorithms for chronic dyspnoea and to assess how accurate and efficient they have been in determining a diagnosis. We undertook this review to inform the need for a validated clinical algorithm incorporated into a Clinical Decision Support System (CDSS) designed for use in primary care.

## Methods

### Inclusion and exclusion criteria

Only full-length peer-reviewed studies (randomised controlled trials, cohort, case-control, cross-sectional studies and systematic reviews) published in English from 1946 to November 2020 were included in this review. We excluded abstracts for which a full-length paper was not available. Study participants were patients of any age who presented to a primary, secondary or tertiary care services with unknown causes of chronic dyspnoea (duration ≥1 month). The main outcome of interest was the use and diagnostic accuracy of an algorithmic approach to the assessment of dyspnoea.

### Search strategy, study selection and analysis

A comprehensive MEDLINE search using the MESH terms “dyspnea/laboured breath/breath short/breathlessness” and “decision support system, clinical/diagnosis computer-assisted/decision support techniques/medical decision making” was conducted. Secondary searches were performed using EMBASE using the same keywords. Additional literature was identified by searching the citation list of the identified articles. We also looked for relevant literature using Google Scholar. All the searched results were merged into one single Endnote Library and all duplicates were removed. Once duplicates were removed, the investigators (C.J., N.B., N.H. and A.P.S.) independently reviewed the title and abstracts and excluded irrelevant studies. The full-length relevant articles were retrieved and examined to further determine if they met inclusion criteria. Conflicts were resolved through discussion with all investigators. Data were extracted to a specifically designed form that included details on the patient cohort, clinical algorithm and investigations utilised and accuracy of the algorithms. Results were analysed descriptively and presented in a narrative format.

## Results

**Fig. 1:**
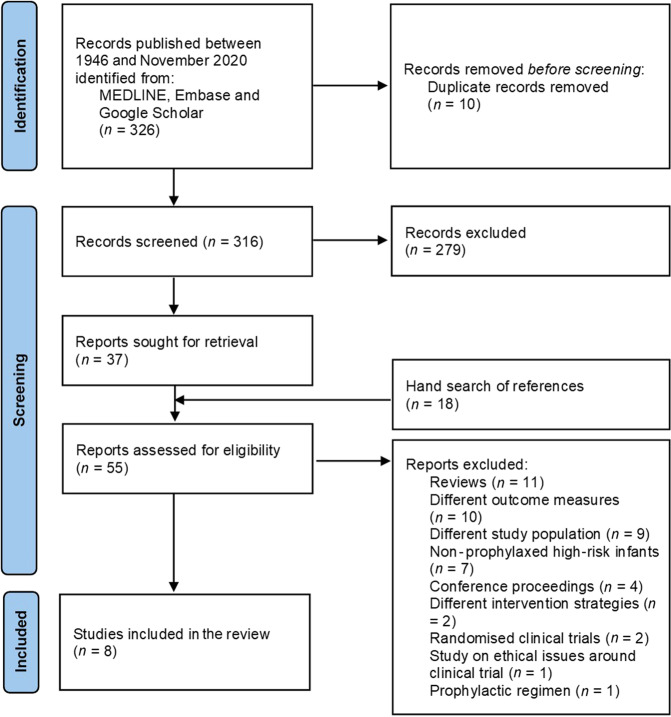
Study selection flow diagram.

The initial search identified 326 abstracts with another 18 papers were extracted from the reference search of the included papers, after removing 10 duplicates there were 316 abstracts for initial review. C.J., N.B., N.H. and A.P.S. independently reviewed all the abstracts. Thirty-seven abstracts were included for full-text review. Another 18 papers were extracted from the reference search of the included papers, making a total of 55 articles reviewed in full length. After further review of the full articles, eight studies were included in the final analyses. (Fig. 1) The primary reasons for exclusion were that the average duration of dyspnoea was of shorter duration than one month, there was an inadequate description of the CDSS or algorithm, the algorithm was not validated and/or there was no quantification of outcome after its use. One study (Pratter et al.^18^) included patients with dyspnoea from >3 weeks but reported a mean dyspnoea duration of 2.9 years (range 3 weeks to 25 years), hence it was decided to include it as part of the review.

### Profile of the studies and the study participants

All eight studies^18–25^ included were primary studies. Patients were recruited from general practice for only one^22^ of the eight primary studies, and from tertiary care services for the other seven studies^18–21,23–25^. The age range of the study participants was 20 to 80 years, and 60% of the study participants were women (1234 women vs 792 men). The duration of dyspnoea was 3 weeks to 25 years (Table 1).

**Table 1:** Characteristics of the included study population and final diagnoses.

Reference	Study population	Sample size	Study setting	Mean (SD)/median (min–max) age in years	Dyspnoea score/severity	Mean duration of dyspnoea in months	Respiratory final diagnosis (%)^b^	Cardiac final diagnosis (%)^b^	Other diagnosis (%)^b^	Remain unexplained (%)^b^
DePaso et al.^19^	Patients with unexplained dyspnoea for >1 month	72^a^	Pulmonary and critical care unit of the clinic	Not mentioned	Not mentioned	Not mentioned	40 (54)	10 (13)	8 (11)	14 (19)
Gumus et al.^20^	Patients with dyspnoea for >1 month	462 (M 172, F 290)	Outpatient Department University Pulmonary Practice Unit	53 ± 17	Not mentioned	Not mentioned	101 (22)	184 (40)	177 (38)	
Huang et al.^25^	Patients with unexplained dyspnoea for >1 month	530 (M 174, F 356)	University Multidisciplinary Dyspnoea Intolerance Center	57 (44–68)	Not mentioned	Not mentioned, 89 patients (16.8%) have dyspnoea complaints >4 years 511 days (292–1095 days)	131 (25)	187 (35)	300 (57)	
Martinez et al.^21^	Patients with unknown cause of dyspnoea, mean of 23 months (range 3–240 months)	50 (M 23, F 27)	Pulmonary and critical care unit of the clinic	55 (26–82)	Not mentioned	Not mentioned	17 (34)	7 (14)	24 (48)	7 (14) diagnosed as normal
Ocal et al.^24^	Patients with unexplained dyspnoea for >1 month	250 (M 124, F 126)	Pulmonary Clinic	59.4 ± 13.2	Not mentioned	Not mentioned	148 (59.2)	155 (62)		42 (17)
Pedersen et al.^22^	Patients aged 60–79 years with dyspnoea grade ≥1 as per WHO for >1 month	129 (M 40, F 89)	General practice	71.5 (60–79)	Mean grade 2	Not mentioned	68 (53)	27 (21)	49 (38)	15 (12)
Pratter et al.^18^	Patients with dyspnoea for at least 3 weeks	85 (M 48, F 37)	University pulmonary practice unit	52 (17–86)	2.93 (BMRC Index), 5.74 (Mahler dyspnoea index), 2.56 (patient self-rating)	2.9 years (3 weeks–25 years)	56 (66)	9 (10.6)	21 (25)	
Pratter et al.^23^	Patients with dyspnoea for >8 weeks	123 (M 48, F 75)	University hospital	60.2 ± 15.1	6 ± 2.3	24.5 ± 33.9	78 (53)	23 (16)	46 (31)	1 (1)

*M* male, *F* female.

^a^Gender proportions not reported.

^b^It must be noted that an individual can be classified as having more than one diagnosis.

### Clinical algorithms for assessment of dyspnoea

In addition to history and physical examination, 32 different types of diagnostic examinations were reported in the studies (Table 2). They ranged from less invasive tests such as spirometry and electrocardiography to bronchoscopy and open lung biopsy. Furthermore, evaluation by psychiatrist, cardiologist and post-disease-specific therapy were included as steps in the assessment process.

**Table 2:** List of investigations used in the studies and the order in which they are utilised for assessment of dyspnoea when available.

Tests	Pedersen et al.^22^	Gumus et al.^20^	Pratter et al.^23^	Pratter et al. 1989^18^	DePaso et al.^19^	Martinez et al.^21^	Huang et al.^25^	Ocal et al.^24^
History		Stage 1	Stage 1	Initial evaluation	Initial evaluation	Initial evaluation	Initial evaluation	Initial evaluation
Physical examination		Stage 1	Stage 1	Initial evaluation	Initial evaluation	Initial evaluation	Initial evaluation	Initial evaluation
Spirometry	Stage 1	Stage 1	Stage 1	Second evaluation in all patients	Initial evaluation	Initial evaluation	Initial evaluation	Second evaluation
Flow volume loop				As needed	As needed			
Lung volume	Stage 3	Stage 3	Stage 1	As needed				
Lung diffusion capacity	Stage 2	Stage 2	Stage 1	As needed	As needed			
Electrocardiogram	Stage 1	Stage 1					Initial evaluation	
Chest X-ray	Stage 3	Stage 1	Stage 1	Initial evaluation	Initial evaluation		Initial evaluation	
Sinus X-ray				As needed				
Full blood count		Stage 1	Stage 1					
Serum haemoglobin	Stage 2							
Thyroid function test/TSH; free T4	Stage 2	Stage 1	Stage 1		Second evaluation in all patients			
Basic chemistries			Stage 1		Second evaluation in all patients			
Brain natriuretic peptide			Stage 1					
Oxygen saturation using pulse oximetry		Stage 1						
Bronchial provocation test		Stage 2	Stage 1	As needed	As needed			
Echocardiogram/stress echocardiography	Stage 2	Stage 2	Stage 3	As needed	As needed		Initial evaluation	Second evaluation
Cardiac MRI	Stage 3							
Cardiopulmonary exercise test	Stage 3	Stage 2	Stage 2	As needed	As needed	Only evaluation	Second evaluation	
CT angiogram	Stage 3	Stage 2						
Chest CT scan			Stage 3	As needed				
Ventilation/perfusion scan		Stage 2	Stage 3		As needed			
Bronchoscopy		Stage 3	Stage 3	As needed				
Open lung biopsy				As needed				
Left cardiac catheterisation		Stage 3	Stage 3					
Right cardiac catheterisation		Stage 3	Stage 3					
Arterial blood gas		Stage 3			Second evaluation in all patients			
Scintigraphy		Stage 2		As needed				
Thoracentesis			Stage 3					
Upper GI endoscopy			Stage 3					
Barium swallow				As needed				
24 h oesophageal pH probe				As needed				
Sinus CT			Stage 3					
Polysomnography				As needed				
Maximal inspiratory pressure (MIP) and maximal expiratory pressure (MEP)/respiratory muscle strength					As needed			
Evaluation by psychiatrist		Stage 2						
Evaluation by cardiologist		Stage 2						
Response to disease-specific therapy				As needed				

The studies found can be classified as three types—those reporting a step-wise assessment process, those advocating a minimum package of tests for all dyspnoea patients followed by clinical judgement in the provision of testing, and another group reporting the utility of cardiopulmonary exercise testing (CPET) for routine assessment of unexplained dyspnoea cases.

### Step-by-step assessment

Three of the included studies^20,22,23^ used a three-step clinical review process to assess dyspnoea. All patients underwent the first stage of screening assessment which comprises history and physical examination^20,23^, and initial non-invasive or routine tests. If a cause of dyspnoea was not established in Stage 1 then patients were assessed using more specialised investigations (Stage 2). Patients for whom the cause of dyspnoea was not ascertained after completion of Stage 2 were then moved to Stage 3 investigations. In each proposed algorithm, Stage 3 included more invasive and expensive investigations. Apart from history and physical examination, the tests that were commonly used for the first stage across the three studies were spirometry, electrocardiography, chest x-ray, thyroid function tests and full blood count (Table 2). Echocardiogram and cardiac exercise/stress test were used commonly in Stage 2, while bronchoscopy and cardiac catheterisation would be undertaken in Stage 3.

At the end of Stage 1, a cause for dyspnoea was ascertained for 35% of the patients. Stage 1 and Stage 2 in combination diagnosed 65% of the patients with dyspnoea, and more than 90% of the dyspnoea cases were diagnosed by a combination of stages one, two and three.

### Package of tests followed by clinical judgement

Two of the studies used a logical flow of investigations based on the discretion of the study pulmonologist. The first by Pratter et al. in 85 patients (median age 52 years) included an initial evaluation comprised of extensive history taking, physical examination, assessment of the severity of dyspnoea and chest roentgenogram^18^. Following this, more advanced investigations included spirometry, lung volume measurement, flow volume loops, bronchial provocation, single-breath diffusing capacity, metabolic exercise test, radionuclide ventriculography and cardiac scan, 24-h oesophageal pH monitoring and CPET. A final diagnostic decision was made by agreement between two expert clinicians, based on these results, which represented the “true” diagnosis. Additionally, the degree of physiologic dysfunction demonstrated on objective testing had to be consistent with the patient’s functional limitation and could not be attributed to another disorder. Response to specific treatment was not required as a diagnostic criterion in those conditions for which specific therapy was unavailable at the time, but for treatable responsive conditions such as asthma, positive treatment response was an additional mandatory criterion.

The initial evaluation provided objective evidence of a clear diagnosis in 65% of patients, primarily identifying COPD, asthma, interstitial lung disease (ILD) or cardiomyopathy. Almost half the final diagnoses were non-respiratory. Physicians’ provisional diagnoses following history, physical examination and chest X-ray were accurate 66% of the time compared with final diagnoses. Even so, this accuracy varied, reaching an 81% accuracy when the cause was asthma, COPD, ILD or cardiomyopathy but falling to 33% for less common causes. In relation to the respiratory diagnoses, bronchoprovocation challenge testing with methacholine had a 95% positive predictive value and a 100% negative predictive value for the diagnosis of asthma. A history of smoking in combination with spirometry was useful in assessing dyspnoea due to COPD, and in this study^18^, no never-smoker had a final diagnosis of COPD. The presence of crackles on physical examination and chest roentgenogram had a high positive predictive value for ILD (79%) and the absence of crackles had a high negative predictive value (98%). Lung volume measurement was not helpful in reaching a diagnosis of ILD in this study. CPET with measurement of gas exchange was particularly helpful in identifying dyspnoea with a psychogenic component or if determined to be due to deconditioning.

In another study undertaken by DePaso and colleagues^19^ in patients with unexplained chronic dyspnoea, an alternative logical diagnostic approach was assessed. The assessment started with taking a targeted history and including age at onset of dyspnoea, duration, pattern and intensity and physical examination. Seventy-two patients with dyspnoea unexplained by a pulmonologist’s repeat history and physical examination, chest X-ray and spirometry made up the final study group and underwent a second more focused history. Those with a negative history had routine biochemistry along with serum thyroxine and arterial blood gas (ABGs) assessment at rest breathing room air. The remaining patients underwent more non-invasive testing, at the specialist physician’s discretion and testing stopped when a diagnosis that explained the dyspnoea was reached. This tier of tests included single-breath carbon monoxide diffusion capacity (DLCO), repeat spirometry, inspiratory flow-volume loop, measurement of maximal inspiratory and expiratory pressures, ventilation-perfusion lung scan, a two-dimensional echocardiogram, cardiac exercise treadmill examination, Holter monitoring, methacholine or exercise bronchoprovocation test, computed tomographic scanning of the thorax (thoracic CT), upper gastrointestinal series, 24-h oesophageal pH monitoring and CPET.

In this study, the diagnosis of the cause of dyspnoea was based on accepted diagnostic criteria^26^, the attributed diseases had to be a known cause of dyspnoea, and treatment of the cause had to result in improvement in dyspnoea. Additionally, determination of cause was verified by a minimum 1 year follow-up period, which failed to reveal any additional diseases known to be associated with dyspnoea. Out of the 72 patients assessed for unexplained dyspnoea, the cause of dyspnoea was explained by respiratory tract diseases in 26 (36%) patients, cardiac diseases in 10 (14%), hyperventilation syndrome in 14 (19%), gastroesophageal reflux in 3 (4%), thyroid disease in 2 (3%), poor conditioning in 2 (3%) and renal diseases in 1 patient. The cause of dyspnoea could not be established in 14 (19%) patients. The duration and intensity of dyspnoea offered no diagnostic insight.

Age at onset of <40 years had 81% positive predictive value and 77% negative predictive value for hyperventilation or bronchial hyperactivity assessed by methacholine bronchoprovocation tests^26^. In addition, age of onset <40 years with P(A-a) O_2_ ≤ 20 mmHg had 89% positive predictive value and 71% negative predictive value for hyperventilation or airways disease characterised by bronchial hyperreactivity. The positive predictive value and the negative predictive value reached 100% and 67% respectively when age at onset of <40 years with P(A-a) O_2_ ≤ 20 mmHg was combined with intermittent dyspnoea. The authors concluded that patients with unexplained dyspnoea and symptom onset aged under 40 years, with P(A-a) O_2_ < 20, were most likely to have hyperventilation or airway hyperresponsiveness. Therefore, the most useful, single non-invasive test when the diagnosis of dyspnoea was uncertain in a young adult, was a bronchial challenge. With the exception of bronchial challenge, the diagnostic value of any other single non-invasive test was poor in this study.

On the other hand, Ocal and colleagues^24^ in a retrospective review of 250 patients with chronic dyspnoea (mean age 59.4 ± 13.2 years) which remained unexplained following clinical evaluation (history and physical examination) by specialists reported the utility of spirometry and transthoracic echocardiography. They showed that 83% of these patients can be diagnosed as having either heart and/or lung disease using only both tests. Importantly, they showed that 95 patients (38%) had a multimorbid cause of dyspnoea wherein they had both heart and lung disease concomitantly. Asthma and COPD, and diastolic heart failure were the most common lung and heart diseases respectively.

Another study^25^ from a multi-disciplinary dyspnoea centre reported the utility of CPET in 864 patients with chronic dyspnoea (median age 57 years). After an initial evaluation using a suite of pulmonary function tests, chest imaging, electrocardiogram, echocardiogram and historical data, 36% of patients received a diagnosis of the underlying cause of their dyspnoea. The remaining 554 unexplained patients underwent a CPET examination, although complete details were available for only 530 patients who were included in the analysis. The study reported that the underlying explanation for dyspnoea was successfully determined in all patients post CPET. Ultimate diagnoses included pulmonary arterial hypertension, heart failure with preserved ejection fraction, dysautonomia, oxidative (mitochondrial) myopathy and primary hyperventilation. A median time of 27 days (13 to 53 days) was reported to obtain this final diagnosis post referral to the multidisciplinary clinic which contrasted to the median of 511 days (292 to 1095 days) with dyspnoea prior to referral.

### Potential role of CPET in assessing unexplained dyspnoea

One of the identified studies investigated only the role of graded, comprehensive CPET in assessing the cause of unexplained dyspnoea^21^ (median age 55 years). Patients with dyspnoea on exertion with no suggestive findings on routine blood examination and chest radiograph and with normal flow-volume loop, an FEV_1_ > 80% predicted, FVC > 80% predicted and FEV_1_/FVC > 0.7; and the ability to complete an adequate symptom-limited CPET were included in the study^21^. In this study CPET results were compared with final clinical diagnosis in 50 patients. In the majority of patients (*n* = 24) the CPET study was suggestive of poor conditioning but could not exclude a cardiac cause. Of these, 14 patients responded to exercise training and/or weight loss, 3 had cardiac disease, 7 had airway hyperresponsiveness, and 4 had psychogenic dyspnoea. In 13 patients with normal CPET results, the cause of dyspnoea was assessed as gastroesophageal reflux in 1, hyperactive airway disease in 2, psychogenic dyspnoea in 4, and no identifiable disease in 6. The authors concluded that CPET is useful in identifying a cardiac or a pulmonary cause but has limited sensitivity in distinguishing cardiac cause from deconditioning. Subsequent studies, however, suggest that cardiac disease and deconditioning can be distinguished more readily with CPET^27^. When dyspnoea is unexplained after clinical history and examination, lung function testing, chest X-ray and echocardiogram, CPET remains a highly informative test^28^.

## Discussion

This literature review revealed the scarce research that has been undertaken to help clinicians develop an accurate and efficient approach to the diagnosis of dyspnoea. The research we report here has, however, demonstrated that a stepwise approach to dyspnoea assessment that starts with simple and then more expensive or invasive tests only as the initial steps fail, could achieve an accurate diagnosis in the majority of patients^29^.

Our review suggests detailed history taking, physical examination, full blood count along with spirometry, chest X-ray and electrocardiogram are the most appropriate initial clinical assessments to establish a cause of dyspnoea (>30%). While history taking and physical examination were reported to be essential components in all studies, no study aimed to validate a high yield approach to it which is important considering the time constraints in primary care. Even so, several expert reviews on history taking and physical examination for chronic breathlessness such as the Breathing SPACE framework^30^, IMPRESS framework^31^ and a review by Baxter et al.^32^ from the Primary Care Respiratory Society are available as references for clinical practice.

History taking and physical examination may help direct initial investigations if the clinical presentation aligns with well-recognised clinical diagnoses; however, spirometry, full blood count or electrocardiogram, easily arranged within a primary care setting, can readily inform a less clear presentation. Full blood counts can not only support elucidating the various causes of anaemia but also myeloproliferative disorders and other pathologies. The Tefferi et al.^33^ review for interpreting and pursuing abnormal full blood counts provided greater depth in describing these various possible combinations of full blood count results and its potential pathologies.

Subsequent appropriately directed tests include an echocardiogram, thoracic CT, lung volumes and DLCO. When combined with other more specialised tests such as CPET, CT angiogram, ABGs and bronchoscopy it was reported that a diagnosis can be established in the majority of patients presenting with dyspnoea (~90–100%). We note that some of these investigations are only available in secondary and tertiary care with specific use cases. ABGs for example were used in the two studies that reported them only in patients with concomitant hypoxia (oxygen saturation <95%) or utilised to measure the alveolar to arterial (A-a) oxygen gradient which was found to support diagnosis of functional dyspnoea in patients aged <40 years. It is, however, worth noting that since several of these studies were published, several tests investigations have become much more practical in primary care (e.g. oxygen saturation measurements), or very readily accessed (e.g. thoracic CT imaging with reports).

**Fig. 2:**
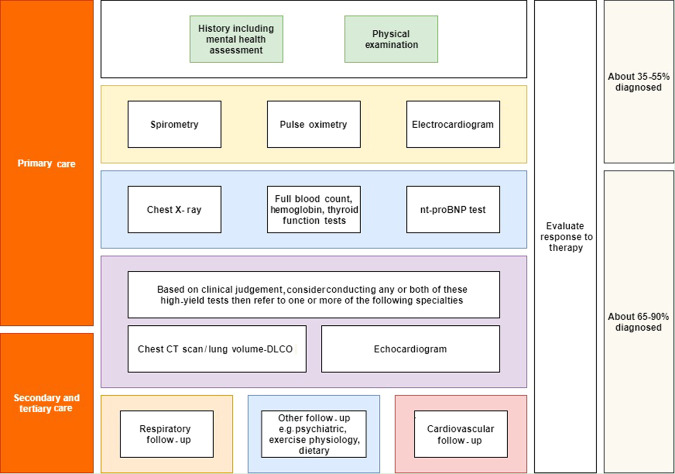
A summary of the stepwise approach for dyspnoea assessment and the probability of elucidating the causal diagnosis based on the included studies. DLCO diffusing capacity of the lungs for carbon monoxide.

A stepwise approach to assess dyspnoea based on a summary of the general consensus from included studies, possible utility in primary care and their possible diagnostic yield could be found in Fig. 2.

As a symptom that manifests in many different diseases across respiratory, cardiovascular, musculoskeletal, mental health and metabolic conditions, dyspnoea can be particularly difficult and time consuming to assess in primary care, where it typically first presents. It is also low on the radar of many people and their health providers despite its serious impact on quality of life & wellbeing. Additionally, patients have their own explanations for it, often blaming themselves for lack of fitness, sedentary lifestyle, smoking or obesity. Nihilism and lack of vigilance on a clinician’s part can also delay diagnosis and the implementation of effective treatment^6–8^. As Ocal and colleagues^24^ had also noted, multimorbid causes of dyspnoea are common in practice and must be taken into account during evaluation. Even where the chronic heart and/or lung disease is present, dyspnoea was strongly associated with preventable, addressable lifestyle factors such as physical inactivity, obesity, anxiety and depression^34,35^.

As presented in Fig. 2, spirometry plays one of the most important roles in elucidating the cause of dyspnoea after history and physical examination in practice. Although it is non-invasive and can be readily performed in primary care, many previous studies have shown that spirometry is not routinely utilised in primary care or is performed with sub-optimal technical quality and interpretation^14,15^. White and colleagues in an observational study of spirometry in 6 general practices in the United Kingdom (UK) reported that 15% of spirometry test results were incomplete and 40% of those complete were unacceptable by specialist standards^36^. In a more recent validation study in the UK on the validity and interpretation of spirometry recordings in primary care for diagnosing COPD it was reported that while 98.6% of spirometry recordings were of adequate quality according to chest physicians, only 72.5% of spirometry traces labelled as COPD were consistent with obstruction^37^. In Australia, a study in New South Wales reported even lower values with only 57.8% of COPD patients diagnosed with no prior testing in primary care having had post-bronchodilator spirometry results consistent with COPD or asthma^38^. These studies demonstrate that not only is the quality of recording a problem in some sites but even when of technically adequate quality, interpretation may be inaccurate. This is a situation where a decision support system can help by providing support in both performing the spirometry and its automated interpretation.

In a randomised controlled trial on the validity of remote spirometry performed online via teleconference, it was reported that there were no significant differences in quality found between the online and conventional spirometry values recorded^39^. A study in Italy of 937 GPs on the use of tele-spirometry (diagnosis is performed by a remote specialist) demonstrated that during 2 years in over 20,000 tests, 70% of patients met the criteria for good or partial cooperation and the rate of tele-spirometries that could not be evaluated was low at 9.2%^40^. Although in both these studies there was remote real-time hospital support to guide spirometry taking and interpretation, they illustrate the potential of remote support to improve spirometry performance and interpretation in primary care. Similar systems guided by an automated CDSS system can be developed.

Outside of supporting spirometry, CDSS that are well designed and implemented have the potential to improve health care quality, increase efficiency, improve clinical workflow and reduce health care costs. Globally, studies evaluating the provision of care by clinicians suggest that evidence-based care was delivered 40–55% of the time^18,20,22^. Clinicians require tools to ensure cost and time-efficient diagnosis of dyspnoea, achieving the highest probability of an accurate diagnosis with the lowest number of steps and tests. The majority of systematic reviews have shown that CDSS are effective at reducing these evidence-practice gaps in various chronic conditions such as diabetes and cardiovascular risk factor management^41–43^, and hence could be well suited to assessing and diagnosing dyspnoea.

Our review is limited by the very few studies that have been undertaken over a 30-year period, and in different secondary care settings that already represent a decision that the problem is most likely cardiac or respiratory. Additionally, over this period, access to imaging and sophisticated testing has evolved rapidly. Ease of access, however, can result in a battery of tests being undertaken, rather than a systematic approach that maximises efficiency and minimises costs.

The results suggest that a simple, inexpensive and evidence-based approach to dyspnoea assessment reachable to primary care physicians can lead to an accurate diagnosis in most patients. When dyspnoea remains unexplained, the results also suggest that a specialist referral for further testing can elucidate the causal diagnosis in almost all patients. Incorporating a validated diagnostic algorithm into a CDSS could facilitate a “fast track” to diagnosis and avoid unnecessary tests and consultations. If tested and implemented in primary care and linked to an evidence-based approach to management, diagnostic delays could be avoided, and patient outcomes enhanced.

## Data Availability

The authors declare that all data supporting the findings of this study are available within the paper.
